# Applications of Novel Biodegradable Polymeric Materials

**DOI:** 10.3390/ma15238411

**Published:** 2022-11-25

**Authors:** Joanna Rydz, Marta Musioł

**Affiliations:** Centre of Polymer and Carbon Materials, Polish Academy of Sciences, 41-819 Zabrze, Poland

Commonly used traditional polymeric materials have many advantages, although their resistance to biological agents causes a negative impact on the environment. Therefore, the use of (bio)degradable polymers with a minimal carbon footprint should become widespread due to the growing interest in sustainability, organic recycling, environmental issues, and healthcare. From the sustainability perspective, (bio)degradable polymers represent an interesting and fairly versatile alternative to conventional polymers. There is also increasing demand for (bio)degradable polymers that have been designed as materials for multi-faceted applications with a specific lifetime. Currently, there are challenges related to the design of materials that are stable in use and at the same time susceptible to microbial attack during organic recycling. Materials intended for specific applications must not only perform specific functions but also meet acceptable standards of safety during use and exhibit both chemical and physical stability [1]. An interesting issue in the field of the lifecycle of polyester materials is tests in real conditions in which the actual degradation time can be verified. These studies should take into account climatic and environmental conditions, including, for example, soil composition, and the degradation time may even be up to several years, depending on the structure of the starting material and environmental humidity [2,3,4].

These challenges are driving many scientists around the world to focus their research in this area. This has led to the development of many technologies for obtaining (bio)degradable polymers from both renewable and petrochemical raw materials. Various natural, bio-based, and synthetic (bio)degradable polymers such as poly(1,4-butylene adipate-*co*-1,4-butylene terephthalate) (PBAT), polylactide (PLA), poly(3-hydroxybutyrate) (P3HB), and poly(butylene succinate) (PBS) are now used in many fields. Although consumers are changing their behavior and increasingly expect ecoproducts, the broad introduction of the (bio)degradable polymeric materials to the market must be preceded by a number of other changes, such as improving composting infrastructure, the development of new technology, financial capacity, and bioplastics- and (bio)degradable-polymer-related policies. Nevertheless, in the coming years, an increase in (bio)degradable polymer applications can be expected, as the development of new possibilities in different areas is predicted to lead to an increase in the market for them and also for bio-based materials such as natural fillers, whose addition to compositions with (bio)degradable polymers is intended to improve the properties of the materials, as well as reduce prices [5,6].

The need to reduce the cost of the materials from (bio)degradable polymers forces the development of research towards the creation of biocomposites with cheaper fillers. However, maintaining a high-quality material with repeated mechanical properties is quite a challenge. Therefore, when choosing natural fibers as fillers for biocomposites, one should take into account not only their geometric features, but also, e.g., the growth conditions of the plants they come from. The repeatability of properties may also accompany biocomposites in which waste from, e.g., the agricultural industry, is used as a filler [7]. Since the additives can be made using post-consumer wood resulting from the processing of wood products, the reuse of secondary raw materials for the production of biocomposites can be an environmentally friendly way to minimize and/or manage the amount of solid waste. Bioactive materials, which contain small amounts of antimicrobial additives, are also very attractive solutions for the packaging industry [8]. The specificity of (bio)degradable polymers, their interactions with the additives or fillers used, and the variety of degradation mechanisms, depending on the environment, extend the possibilities of their applications in niche areas.

The use of (bio)degradable polymers, especially in medical applications, requires a proper understanding of their properties and behavior in different environments. Structural elements made of such polymers can be exposed to changing environmental conditions, which can cause defects. Although the degradation of (bio)degradable polyesters has been extensively reported, less information is available on the degradation of a large group of copolymers with various types of degradable and non-degradable building blocks such as water-soluble poly(ethylene glycol) (PEG), poly(propylene glycol) (PPG), and polyvinylpyrrolidone (PVP) that modulate biodegradability of polyesters by adjusting the hydrophilicity. Such copolymers are also electrically neutral at all pH values, which is why it is so important to determine the effect of, for example, processing conditions on polymer properties and also their subsequent behavior during degradation [9,10]. In broadly understood healthcare, (bio)degradable polymers are used not only as a medical device, but also in the treatment process itself, enabling appropriate control of drug delivery.

In recent years, there has been considerable development in both synthetic and natural drug-release materials for use in drug-delivery systems. Most such systems are based on synthetic polymers, such as polyesters, polyphosphazene, or poly(lactide-*co*-glycolide) (PLGA), due to their desirable pharmacokinetics and controllable hydrolytic degradation profiles [11]. Although safe, their properties and processing requirements limit their use in certain areas of sustained delivery, such as protein therapeutics, where product stability may be an issue. Silk fibroin is a biocompatible, non-toxic, mechanically tough protein used as a material for biomedical applications, which has also gained particular interest as a drug carrier vehicle [12]. Drug release systems gaining great popularity in medicine open up new possibilities for their use also outside this area.

Conventional agrochemical formulations can pollute the environment, particularly in the case of intensive cultivation. Additionally, in modern agriculture, there is a growing tendency to use environmentally friendly release systems as carriers of active substances, which are aimed to improve efficiency and limit the cost of application and minimize negative environmental effects. The active substance should be gradually released from the (bio)degradable polymer matrix throughout the growth season of plants at a level that ensures optimal growth of crop plants while destroying the target weeds. At the same time, environmentally friendly polymeric carriers should be biodegradable in soil without creating non-degradable residues [13,14,15].

This Special Issue aims to provide a contemporary overview of recent developments in the field of (bio)degradable polymer or composites applications covering aspects of the current trends in the expansion of polymer material applications.

## Data Availability

Not applicable.
